# Delivering brief physical activity interventions in primary care: a systematic review

**DOI:** 10.3399/BJGP.2021.0312

**Published:** 2021-11-16

**Authors:** Louise H Hall, Rachael Thorneloe, Rocio Rodriguez-Lopez, Adam Grice, Mangesh A Thorat, Katherine Bradbury, Meghana Wadnerkar Kamble, Grace N Okoli, Daniel Powell, Rebecca J Beeken

**Affiliations:** National Institute for Health Research (NIHR) in-practice fellow;; Centre for Behavioural Science and Applied Psychology, Sheffield Hallam University, Sheffield.; National Institute for Health Research (NIHR) in-practice fellow;; National Institute for Health Research (NIHR) in-practice fellow;; Centre for Cancer Prevention, Wolfson Institute of Preventive Medicine, Barts, and The London School of Medicine and Dentistry, Queen Mary University of London, London.; NIHR Southampton Biomedical Research Centre, NIHR Applied Research Collaboration Wessex, Southampton.; School of Health Sciences, University of East Anglia, Norwich.; Institute of Population Health Sciences, Barts, and The London School of Medicine and Dentistry, Queen Mary University of London, London.; Institute of Applied Health Sciences, University of Aberdeen, Aberdeen.; Leeds Institute of Health Sciences, University of Leeds, Leeds.

**Keywords:** brief interventions, disease prevention, health promotion, physical activity, primary care, systematic review

## Abstract

**Background:**

Physical activity (PA) brief interventions (BIs) involving screening and/or advice are recommended in primary care but frequency of delivery is unknown.

**Aim:**

To examine the extent to which PA BIs are delivered in primary care, and explore factors associated with delivery, receipt, and patient receptivity.

**Design and setting:**

A mixed-methods systematic review of studies conducted worldwide, with a narrative synthesis of results.

**Method:**

CINAHL, EMBASE, MEDLINE, and APA PsycINFO index databases were searched for qualitative and quantitative studies, dating from January 2012 to June 2020, that reported the level of delivery and/or receipt of PA BIs in primary care, and/or factors affecting delivery, receipt, and patient receptivity. Quality was assessed using the Mixed Methods Appraisal Tool. Attitudes towards and barriers to delivery were coded into the Theoretical Domains Framework and the Capability, Opportunity, and Motivation Behaviour model.

**Results:**

After screening a total of 13 066 records, 66 articles were included in the review. The extent of PA screening and advice in primary care varied widely (2.4%–100% and 0.6%–100%, respectively). PA advice was delivered more often to patients with a higher body mass index, lower PA levels, and/or more comorbidities. Barriers — including a lack of time and training/guidelines — remain, despite recommendations from the World Health Organization and National Institute for Health and Care Excellence that PA advice should be provided in primary care. Few studies explored patients’ receptivity to advice.

**Conclusion:**

PA BIs are not delivered frequently or consistently in primary care. Addressing barriers to delivery through system-level changes and training programmes could improve and increase the advice given. Understanding when patients are receptive to PA interventions could enhance health professionals’ confidence in their delivery.

## INTRODUCTION

Physical inactivity is a global public-health problem.^1^^,^^2^ In the UK, levels of inactivity are increasing: approximately 32% of males and 36% of females failed to meet the government’s physical activity (PA) recommendations in 2018.^3^ Physical inactivity increases the risk of poor physical and mental health, is estimated to account for as many deaths in the UK as smoking (one in six), and costs the NHS around £0.9 billion annually.^4^

In its *Global Recommendations on Physical Activity for Health*, the World Health Organization suggests PA advice should be provided in primary care.^5^ Correspondingly, in the UK, the National Institute for Health and Care Excellence (NICE) recommends that primary care practitioners deliver brief PA advice to patients who are not currently meeting PA guidelines.^6^ NICE defines brief advice as *‘verbal advice, discussion, negotiation or encouragement, with or without written or other support or follow-up’*.^6^

Previous reviews have found brief interventions (BIs) to be effective at increasing (self-reported) PA in the short term, with some evidence that this can be maintained in the longer term (that is, 12 months).^7^^,^^8^ However, barriers to giving and receiving PA advice in primary care are rife; a review in 2012 reported a variety of barriers, including lack of resources and perceived (in)effectiveness of advice.^9^ Since that review was published, the population’s PA levels have not substantially increased,^10^ despite various initiatives nationally and globally to increase PA advice delivered in primary care.^11^^,^^12^ Additionally, the UK’s recent GP workforce crisis^13^^,^^14^ may have impacted GPs’ capacity to include PA discussions in consultations. Thus, an updated review on barriers and facilitators to delivering PA advice in primary care is warranted. Furthermore, little is known about how often, and to whom, this advice is given. This knowledge is crucial for understanding how PA BIs are implemented in practice and identifying potential areas for improvement. The aim of this mixed-methods systematic review was to:

- examine the extent to which PA BIs (PA screening and/or advice) are delivered in primary care; and
- explore factors associated with delivery, receipt, and patient receptivity.

**Table 1:** How this fits in

Physical activity (PA) brief interventions delivered in primary care consultations can increase levels of PA in the general population, but there is a lack of understanding regarding the frequency of, and factors associated with, delivery. This review reports high variation in the frequency and context of delivery and receipt, and outlines common barriers to and facilitators of (coded in the Theoretical Domains Framework and Capability, Opportunity, and Motivation Behaviour model) practitioner delivery. Identified barriers could be addressed through system-level changes, improved educational resources, and training to increase practitioner knowledge and confidence, and subsequently improve patient receptivity and PA uptake.

## METHOD

### Search strategy

Literature index databases were searched for quantitative articles reporting the level of delivery and/or receipt of PA BIs in primary care consultations for health promotion/disease prevention, and quantitative/qualitative articles reporting factors affecting delivery, receipt, and patient receptivity. In July 2018, and again in July 2020, an information specialist carried out separate searches of the Cumulative Index to Nursing and Allied Health Literature (CINAHL), EMBASE, MEDLINE, and American Psychological Association (APA) PsycINFO databases; Supplementary Box S1 provides example search terms for PsycINFO. The review was prospectively registered on PROSPERO, an international prospective register of systematic reviews (reference: CRD42018103812).

### Article selection and data extraction

Two authors screened the titles and abstracts using the specified inclusion criteria (outlined in Supplementary Box S2) and erring on the side of inclusion. Three authors then reviewed 20% of the titles and abstracts to ensure reliability. In all, 20% of the full-texts were double-screened by two authors, and disagreements were arbitrated by a third author. References of included articles were hand searched for additional eligible studies.

In total, 100% of the data were extracted in duplicate by four independent authors using an electronic spreadsheet. Discrepancies were checked by another reviewer. Key study characteristics are given in Supplementary Table S1, and the main outcomes of patient and practitioner receipt/delivery of PA BIs (levels of screening and advice) are outlined in Supplementary Tables S3 and S4.

### Quality assessment

Study quality was assessed by one reviewer using the Mixed Methods Appraisal Tool;^15^ 20% of studies were assessed by a second reviewer and checked for consistency.

### Analysis

In order to examine the extent to which PA BIs are delivered in primary care, quantitative data were extracted on the reported frequency of:

- PA screening;
- delivery of PA advice by health professionals; and
- patient-reported receipt of PA BIs.

A quantitative synthesis of these data was not possible, because of large heterogeneity in the definition and measurement of PA BIs. A narrative synthesis was, therefore, conducted.

In order to explore factors associated with delivery, receipt, and patient receptivity, quantitative data were extracted inductively from articles, in duplicate, by four reviewers; these were then coded as either patient or health professional/system factors. Qualitative data on health professionals’ attitudes and perceived barriers towards delivery, as well as patients’ views, attitudes, and receptivity towards PA BIs, were extracted inductively from the articles using the articles’ own phrasing/codes. Similar codes were grouped together by one reviewer who has expertise in behaviour change theory. Codes relating to health professionals’ attitudes or barriers were mapped onto the Theoretical Domains Framework (TDF) and Capability, Opportunity, and Motivation Behaviour (COM-B) model by that same reviewer and one other to assist in the identification of key components for future interventions aiming to increase PA BI delivery.

## RESULTS

**Figure 1.**
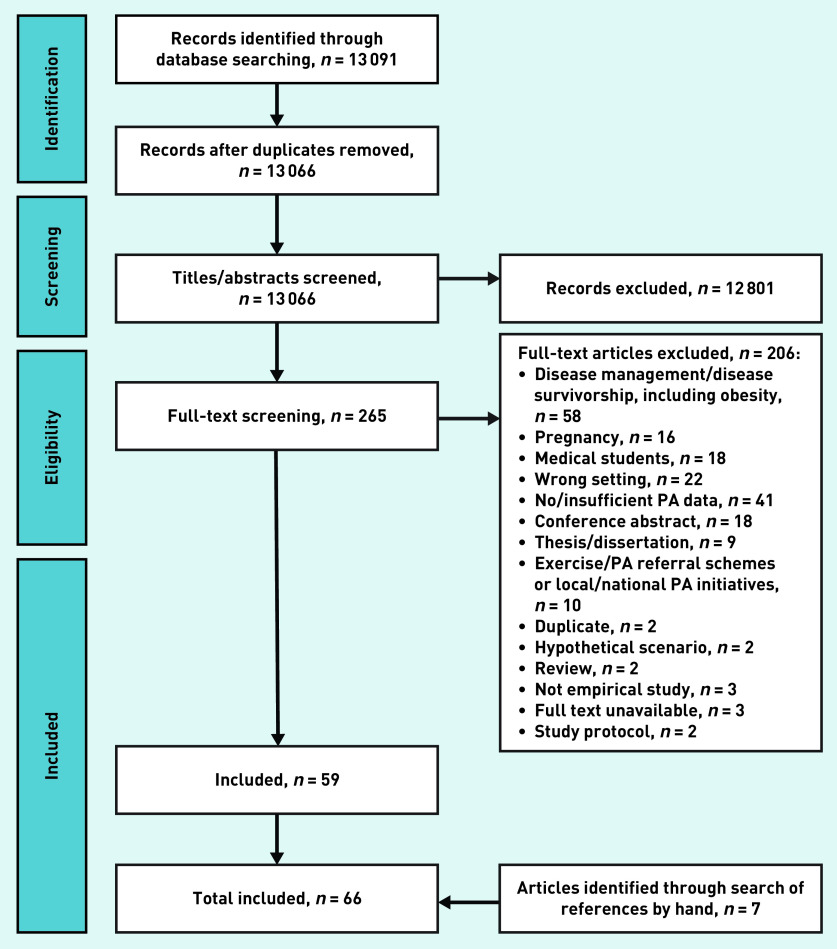
*Flow diagram of article search and inclusion process.* *PA = physical activity.*

The database searches identified 13 066 records, once duplicates were removed (Figure 1), and 59 eligible articles.^16^^–^^74^ Hand-searching references identified seven further studies,^75^^–^^81^ giving a total of 66 articles that could be included in the review. The majority of studies (*n* = 40) included data collected from health professionals,^17^^–^^22^^,^^25^^,^^26^^,^^28^^–^^30^^,^^33^^,^^34^^,^^36^^,^^37^^,^^39^^,^^42^^–^^46^^,^^49^^,^^50^^,^^56^^–^^59^^,^^65^^,^^66^^,^^69^^–^^72^^,^^75^^–^^81^ and used cross-sectional surveys (*n* = 54).^16^^–^^19^^,^^21^^,^^22^^,^^27^^–^^34^^,^^36^^–^^61^^,^^63^^–^^68^^,^^71^^,^^73^^,^^74^^,^^77^^–^^81^ Many studies (*n* = 32) were conducted (in whole or part) in North American populations (Supplementary Table S1).^16^^,^^22^^,^^24^^–^^27^^,^^31^^,^^33^^,^^35^^,^^38^^,^^39^^,^^42^^–^^46^^,^^48^^,^^50^^,^^52^^,^^53^^,^^56^^,^^57^^,^^62^^,^^63^^,^^66^^,^^68^^,^^71^^–^^74^^,^^77^^,^^78^

### Quality assessment

The majority of studies were of moderate quality. Most quantitative descriptive studies used appropriate statistical analyses (94%) and appropriate measurements (81%), many of which were pilot tested, and/or developed using Delphi methods or in consultation with key stakeholders. The risk of non-response bias and the representativeness of the target population was either unclear or inadequate in around half of these studies (Supplementary Table S2).

### Level of PA screening by health professionals

**Figure 2.**
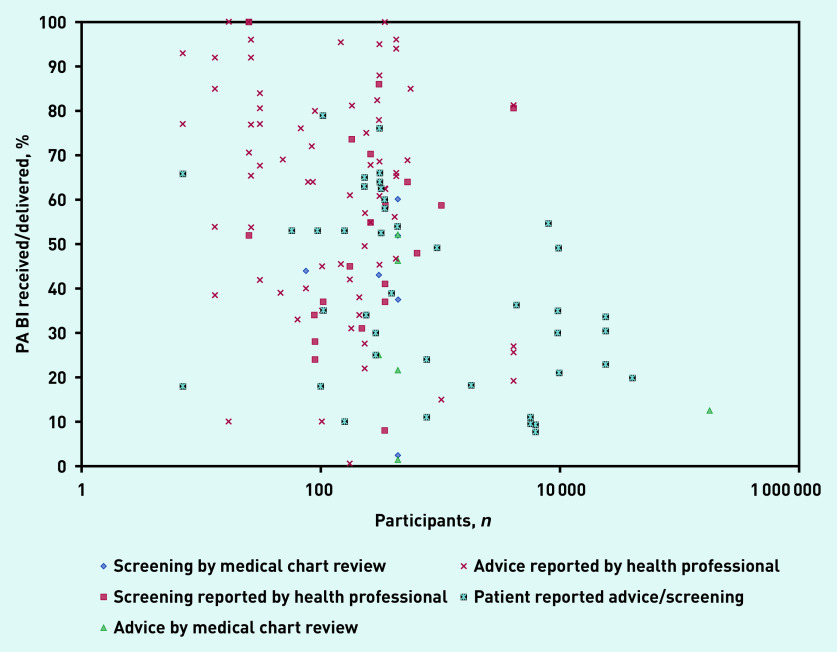
*Percentage of patients receiving physical activity brief interventions in primary care.* *BI = brief intervention. PA = physical activity.*

Eleven studies reported the level of PA screening by practitioners (Figure 2; Supplementary Table S3).^22^^,^^28^^,^^30^^,^^36^^,^^37^^,^^42^^,^^43^^,^^50^^,^^56^^,^^58^^,^^78^ Data from medical chart audits in one medium-quality study reported that the proportion of patients who had their PA levels assessed ranged, depending on appointment type, from 2.4% (unplanned visits) to 60.1% (annual visits) (median 43.5%).^22^ The proportion of practitioners who reported assessing PA for at least some of their patients ranged from 8%^28^ to 100% (median 50%).^78^

### Level of brief PA advice by health professionals

Thirty-one studies (reported in 32 articles) presented the extent to which practitioners provided PA advice or counselling (Figure 2; Supplementary Table S3).^17^^–^^19^^,^^21^^,^^22^^,^^24^^,^^26^^,^^28^^–^^30^^,^^34^^,^^36^^,^^37^^,^^39^^,^^43^^–^^46^^,^^49^^,^^56^^–^^58^^,^^65^^,^^66^^,^^70^^,^^71^^,^^75^^,^^76^^,^^78^^–^^81^ The proportion of practitioners who reported delivering PA advice/counselling ranged from 0.6%^21^ to 100% (median 64.0%).^78^^,^^80^ One high-quality study analysed audio-taped consultations and reported (in two articles) that PA was discussed in 72% of patient visits.^24^^,^^26^ In contrast, the proportion of patients who were given PA advice/counselling, as determined by medical chart audit (in one medium-quality study), ranged, depending on appointment type, from 1.5% (unplanned visits) to 52.2% (annual visits) (median 23.3%).^22^

### Patient-reported receipt of PA BI

Twenty-five studies (reported in 26 articles) provided data on patient receipt of PA BI (Figure 2; Supplementary Table S4).^16^^,^^24^^,^^27^^,^^31^^–^^33^^,^^38^^,^^40^^,^^41^^,^^43^^,^^44^^,^^47^^,^^48^^,^^51^^–^^55^^,^^60^^,^^61^^,^^63^^,^^64^^,^^67^^,^^68^^,^^74^^,^^76^ The proportion of patients reporting that they had received PA advice ranged from 7.7% (of females, 9.4% of males) to 76% (median 35%),^40^^,^^63^ with 13 studies reporting that <40% of patients recalled receiving PA advi ce.^16^^,^^31^^,^^38^^,^^40^^,^^41^^,^^47^^,^^48^^,^^51^^,^^53^^,^^54^^,^^60^^,^^64^^,^^67^ Reviewing audio-taped discussions highlighted that 21% of patients could not accurately recall PA discussions that occurred.^24^

### Factors associated with the delivery or receipt of PA BI

#### Patient factors

Twenty-three studies (reported in 24 articles) examined patient factors associated with PA BI (Supplementary Table S5).^16^^,^^17^^,^^22^^,^^27^^,^^36^^,^^38^^,^^40^^,^^41^^,^^44^^,^^45^^,^^47^^,^^52^^–^^54^^,^^60^^,^^61^^,^^63^^,^^64^^,^^67^^,^^68^^,^^73^^,^^74^^,^^80^^,^^81^ Although the majority of evidence was mixed and inconclusive, the following patient factors were most consistently reported to be significantly and positively associated with the delivery or receipt of PA BI:

- high patient body mass index (*n* = 12);^16^^,^^38^^,^^40^^,^^45^^,^^47^^,^^53^^,^^54^^,^^60^^,^^63^^,^^67^^,^^68^^,^^73^
- physically inactive/sedentary patients (*n* = 6);^38^^,^^47^^,^^53^^,^^54^^,^^60^^,^^67^
- poorer health/greater number of comorbidities (*n* = 5);^38^^,^^53^^,^^60^^,^^67^^,^^73^ and
- greater number of physician visits (*n* = 4).^16^^,^^44^^,^^45^^,^^53^

Patient sex^22^^,^^38^^,^^40^^,^^60^^,^^61^^,^^64^^,^^68^^,^^73^ and age^22^^,^^36^^,^^38^^,^^40^^,^^60^^,^^61^ were often found *not* to be associated with PA BI.

#### Health professional/system factors

Twenty-four studies examined practitioner/system factors associated with PA BI (Supplementary Table S6).^17^^–^^19^^,^^22^^,^^28^^–^^30^^,^^34^^,^^36^^,^^37^^,^^42^^,^^45^^,^^47^^–^^49^^,^^55^^,^^57^^,^^60^^,^^61^^,^^67^^,^^71^^,^^77^^,^^79^^,^^81^ The majority of findings were inconsistent, except the following two points:

- female practitioners were more likely than male practitioners to assess PA (but not necessarily advise);^17^^,^^22^^,^^30^^,^^42^^,^^49^ and
- practitioners with higher levels of PA themselves,^19^^,^^29^^,^^42^^,^^79^ and practitioners with positive beliefs about their capabilities and/or efficacy,^22^^,^^30^^,^^37^^,^^42^ were more likely to deliver PA BI.

### Health professionals’ attitudes and perceived barriers towards PA BI

Twenty-six quantitative^17^^–^^19^^,^^22^^,^^28^^,^^30^^,^^33^^,^^34^^,^^36^^,^^37^^,^^39^^,^^42^^,^^43^^,^^46^^,^^49^^,^^50^^,^^56^^–^^59^^,^^65^^,^^69^^,^^71^^,^^78^^,^^80^^,^^81^ and two qualitative studies^20^^,^^70^ examined health professionals’ attitudes towards delivering PA BI. These were coded into the TDF^82^ and COM-B^83^ model (Supplementary Table S7).

#### Capabilities (psychological)

Twenty quantitative^17^^–^^19^^,^^28^^,^^30^^,^^36^^,^^37^^,^^39^^,^^42^^,^^43^^,^^46^^,^^56^^,^^57^^,^^59^^,^^65^^,^^69^^,^^71^^,^^78^^,^^80^^,^^81^ and one qualitative study^70^ reported barriers and facilitators that were coded under psychological capabilities; of these, 19 studies reported attitudes that fit into the TDF domain of ‘knowledge’.^17^^–^^19^^,^^28^^,^^30^^,^^36^^,^^37^^,^^39^^,^^46^^,^^56^^,^^57^^,^^59^^,^^65^^,^^69^^–^^71^^,^^78^^,^^80^^,^^81^ In 12 of these,^17^^,^^19^^,^^37^^,^^39^^,^^56^^,^^57^^,^^59^^,^^69^^,^^70^^,^^78^^,^^80^^,^^81^ health professionals reported a personal lack of knowledge or training as a barrier to providing PA BI, with a request for additional training also mentioned.^46^ However, the majority of health professionals in six studies perceived they had sufficient knowledge or skills.^18^^,^^30^^,^^36^^,^^39^^,^^46^^,^^65^ In two out of four studies that were coded under the TDF domain of ‘skills’, practitioners reported having difficulty advising patients or including advice in their appointments.^30^^,^^57^

#### Opportunity (physical)

Fifteen quantitative^17^^–^^19^^,^^28^^,^^37^^,^^39^^,^^42^^,^^46^^,^^49^^,^^56^^,^^57^^,^^59^^,^^69^^,^^78^^,^^80^ and two qualitative studies^20^^,^^70^ measured attitudes that were coded under the TDF domain of ‘environmental context and resources’, and the COM-B model category of ‘physical opportunity’. The most commonly cited barriers within these themes were:

- perceived time constraints for including PA discussions in consultations (*n* = 17);^17^^–^^20^^,^^28^^,^^37^^,^^39^^,^^42^^,^^46^^,^^49^^,^^56^^,^^57^^,^^59^^,^^69^^,^^70^^,^^78^^,^^80^ and
- a perceived lack of local services or places to which patients could be referred (*n* = 8).^17^^,^^20^^,^^28^^,^^37^^,^^49^^,^^56^^,^^69^^,^^80^

Further barriers included:

- perceived (lack of) availability of educational resources for health professionals;^19^^,^^39^^,^^56^
- (lack of) effective tools/information to give to patients;^17^^,^^19^^,^^20^^,^^56^^,^^78^ and
- perceived (lack of) opportunities to follow up on PA advice.^20^^,^^57^^,^^70^

#### Motivation (reflective and automatic)

The most commonly coded TDF category within ‘motivation’ was ‘beliefs about consequences’ (*n* = 19).^17^^,^^18^^,^^20^^,^^28^^,^^36^^,^^37^^,^^39^^,^^42^^,^^46^^,^^49^^,^^56^^–^^59^^,^^65^^,^^69^^,^^71^^,^^78^^,^^80^ Within this domain, the most commonly reported barriers to delivery of PA BI were health professionals’ perceived:

- (lack of) patient interest, motivation, or likelihood of adhering to advice (*n* = 14);^17^^,^^18^^,^^20^^,^^28^^,^^39^^,^^42^^,^^46^^,^^49^^,^^56^^–^^59^^,^^78^^,^^80^
- patient expectation of receiving pharmacological treatment (*n* = 6);^17^^,^^18^^,^^39^^,^^56^^,^^57^^,^^78^ and
- (lack of) effectiveness of PA advice (*n* = 7).^17^^,^^39^^,^^56^^–^^58^^,^^71^^,^^78^

Despite these barriers, most practitioners thought that PA BIs were a part of their role (*n* = 11)^17^^,^^18^^,^^20^^,^^28^^,^^30^^,^^42^^,^^43^^,^^49^^,^^59^^,^^69^^,^^80^ and important (*n* = 7);^36^^,^^37^^,^^42^^,^^65^^,^^69^^,^^71^^,^^78^ the majority felt confident about their capabilities (self-efficacy) in providing PA BIs and supporting behaviour change (*n* = 8^22^^,^^28^^,^^30^^,^^33^^,^^50^^,^^58^^,^^59^^,^^65^ of 13 studies measuring confidence/self-efficacy^20^^,^^22^^,^^28^^,^^30^^,^^33^^,^^34^^,^^36^^,^^39^^,^^50^^,^^57^^–^^59^^,^^65^).

### Patients’ views, attitudes, and receptivity towards PA BIs

Four high-quality qualitative studies^25^^,^^35^^,^^62^^,^^72^ explored patient views and attitudes towards PA advice in primary care. Patients felt they had no regular conversations about PA, and that PA conversations lacked substance. The need for a patient-centred approach with follow-up communication was mentioned and some patients were receptive to PA advice if it was clearly linked to contextual factors, such as the potential to reduce medication or pain. Some patients, however, believed practitioners lacked the confidence and knowledge to deliver PA BI, which influenced their receptivity towards advice. In spite of this, provider motivation and support were viewed as important for behaviour change.

## DISCUSSION

### Summary

This mixed-methods review of 66 studies conducted worldwide suggests high variation in the extent to which PA is discussed with patients in primary care. Key practitioner barriers included a lack of time and training/guidelines, and a perceived lack of patient motivation/adherence to PA advice. Few studies have explored patients’ receptivity to such advice; however, conversations with clear relevance to the patient’s contextual factors (for example, medication) appear to be valued.

### Strengths and limitations

To the authors’ knowledge, this review is the first to report on the prevalence of PA BI in primary care, and to link health professionals’ perceived barriers and facilitators to the COM-B model and TDF. However, only articles written in English were included, because of a lack of translation resources, and only 20% of article screening and quality assessment was conducted in duplicate. As only peer-reviewed, published articles were included, a publication bias may have been present.

This review focuses solely on PA screening and advice; studies that examined specific exercise-referral schemes or prescriptions (including social prescribing) were excluded. Future research may benefit from comparing the frequencies of these. Because of a lack of detail in the articles, it was not possible to code Behaviour Change Techniques, despite a plan to do so in the review protocol. In addition, the large degree of heterogeneity of outcome measures made cross-study and cross-cultural comparisons challenging.

The quality of studies was often reduced by the sample not being representative of the target population (or there being a lack of detail to assess this), and a high risk of non-response bias; as such, caution should be taken when generalising findings. It is possible — especially in the sample of health professionals — that those with a particular interest in PA were more likely to participate; as a result, the prevalence of PA BI reported in this review may be an overestimation.

### Comparison with existing literature

This review provides an update of the literature on provider and patient barriers to delivering/receiving PA advice, following Campbell *et al* ’s (2012) review.^9^ It extends their work through coding provider attitudes and barriers into the TDF and COM-B model. Similar provider barriers were identified — namely, perceived likelihood of patient uptake, lack of resources (for example, time and materials), and health professionals’ confidence and knowledge. Lamming *et al* ’s (2017) umbrella review also reported time as a key practitioner barrier.^7^ It is notable that these barriers remain, despite an increased awareness of the importance of PA and recommendations from the World Health Organization and NICE on the delivery of PA interventions.^5^^,^^6^ There is a clear need to identify meaningful ways to tackle these persistent challenges.

Comparing PA with other behaviour-change discussions indicates that those relating to diet, weight, and smoking are often discussed more frequently, whereas those relating to alcohol are discussed less often.^31^^,^^32^^,^^41^^,^^47^^,^^48^^,^^58^^,^^65^^,^^71^ Furthermore, a survey undertaken in Sweden and the US reported that more patients wanted to receive support on diet, weight, and smoking than on PA.^48^ Therefore, PA discussions could be conducted alongside advice on diet and/or weight to increase delivery frequency and patient receptivity.

### Implications for research and practice

PA BIs were more frequently delivered to patients with a higher body mass index, a greater number of comorbidities, and to those who were physically inactive. Practitioners must, therefore, be cautious not to stigmatise patients when deciding when, and how, to conduct these conversations because, if the patient feels they are being stigmatised, it could have a detrimental effect on their psychological and physical health,^84^ and increase inactivity.^85^

Patients often under-reported receiving PA advice, suggesting that training for health professionals that focuses on delivery skills may be needed to increase patient engagement with advice. Opportunistic PA BIs tailored to what is realistically feasible around each patient’s lifestyle are likely to be most effective.

The parallels between health professionals’ perceived barriers to BIs for PA compared with those for smoking cessation^86^ and obesity^87^ — notably, time constraints, lack of experience, and lack of patient motivation — suggest a cultural shift is desirable to address health professionals giving preventive behaviour change interventions lower priority, compared with disease management (including pharmacotherapy).^88^ Although any attempts to address the physical inactivity epidemic are multifaceted, with a need to engage all stakeholders, primary care professionals have a key role owing to the high frequency of patient contact^89^ and the trust patients put in health professionals.^90^

To address this challenge, health professionals — and GPs in particular — need evidence to realise that behavioural interventions have an important place in holistic patient-centred, evidence-based medicine, and reassurance that patients will engage with, and benefit from, them. Health professionals also need clear interventions to offer, with education at undergraduate and postgraduate level, and mandatory within continuing professional development. The recently launched UK’s Moving Medicine toolkit (https://movingmedicine.ac.uk) may help overcome knowledge and resource barriers. However, a recent study demonstrated that, despite educational training successfully addressing GPs’ barriers to providing opportunistic weight-loss interventions during a trial, after the trial ended, GPs reported the same barriers as pre-trial;^91^ therefore, wider system changes may also be required.

There is limited qualitative research on patient views on receiving PA interventions in primary care, and three of the four studies in this review were limited to samples of adults aged >60 years living in North America.^25^^,^^35^^,^^72^ Research is needed on patient receptivity towards PA discussions in the UK, and among people of a wider age range, to inform practitioner training and increase patient engagement with advice.

Only four studies were UK based,^34^^,^^41^^,^^51^^,^^55^ and all indicated that rates of PA BI are low: 15% of GPs reported delivering PA advice to all patients, 18%–35% of patients reported receiving advice, and 53% of patients reported PA screening. More research is needed in the UK to better understand the prevalence of factors associated with, and barriers to and enablers for, delivering and receiving PA BI in UK primary care.

As current research fails to adequately describe the content of PA interventions, it is not possible to comment on the quality of advice given. Future research would benefit from describing the BI and the context in which it is delivered, using the Behaviour Change Taxonomy^92^ and the Template for Intervention Description and Replication (TIDieR) Checklist.^93^

Prevalence of the delivery and receipt of PA BIs in primary care varies widely, with many studies reporting low levels of delivery/receipt. Health professionals have identified a number of barriers to delivering PA advice, including time, knowledge, and confidence. Addressing these barriers through system-level changes and training programmes could improve the consistency, quality, and frequency of advice given. A better understanding of when patients are most receptive to PA interventions in primary care could enhance the interventions’ effectiveness and increase health professionals’ confidence to discuss PA with their patients.
